# Global evidence for the ecological effects of greening of grey infrastructure: a systematic review protocol

**DOI:** 10.1186/s13750-026-00382-z

**Published:** 2026-03-05

**Authors:** Jessica R. Allen, Louise B. Firth, Melanie J. Bishop, Katherine A. Dafforn, Ferrante Grasselli, Mick E. Hanley, Antony M. Knights, Mariana Mayer-Pinto, Abigail McQuatters-Gollop, Kathryn A. O’Shaughnessy, Francesca Porri, Rebecca K. Smith, Elisabeth M.A. Strain, Anaëlle J. Lemasson

**Affiliations:** 1 School of Biological and Marine Sciences, University of Plymouth, Drake Circus, Plymouth, PL4 8AA UK; 2 School of Biological, Earth and Environmental Sciences, Distillery Fields, University College Cork, Cork, Ireland; 3 School of Natural Sciences, Macquarie University, Sydney, NSW 2109 Australia; 4 School for the Environment, University of Massachusetts Boston, Boston, MA 02125 USA; 5 ARC marine Ltd, 3-4 Vaughan Parade, Torquay, TQ2 5EG UK; 6 Centre of Marine Science and Innovation, School of BEES, University of New South Wales, Sydney, NSW 2052 Australia; 7 Dauphin Island Sea Lab, Dauphin Island, AL USA; 8 South African Institute for Aquatic Biodiversity (NRF-SAIAB), Makhanda, 6139 South Africa; 9 Department of Ichthyology and Fisheries Science, Rhodes University, Makhanda, 6140 South Africa; 10 Department of Zoology, University of Cambridge, Cambridge, UK; 11 Institute for Marine and Antarctic Studies, University of Tasmania, Hobart, TAS 7000 Australia; 12 Centre for Marine Socioecology, University of Tasmania, Hobart, TAS 7053 Australia

**Keywords:** Biodiversity enhancement, Eco-engineering, Environmental evidence, Environmental intervention, Evidence synthesis, Nature-based solutions, Urban ecology

## Abstract

**Background:**

The presence of artificial structures in our marine environments is increasing rapidly, with negative impacts for biodiversity. Greening of grey infrastructure (GGI) - an eco-engineering method applied to the marine context - aims to increase the ecological value of traditional grey infrastructure, while still allowing it to perform its primary human-centric function. GGI is a rapidly increasing field of research, being tested and implemented worldwide by academics, private practitioners, governments, non-governmental organisations (NGOs), amongst others, using a variety of methods. Outcomes vary widely, and results are communicated across a range of peer-reviewed and grey literature, rendering the evidence base for the effectiveness of GGI fragmented. To inform future decision-making regarding GGI application, it is critical to consolidate and evaluate existing research. To do so, we propose a systematic review and meta-analysis that will answer the following primary question: “What are the effects of GGI interventions applied to marine structures on the diversity, abundance, biomass, composition, and functional diversity of species on or around these ecologically enhanced structures?”. Additionally, we will answer a series of secondary questions relating to intervention type, material use, geographic variations and other relevant associated variables.

**Methods:**

This systematic review will follow the Collaboration for Environmental Evidence Guidelines and Standards for Evidence Synthesis in Environmental Management. Using a defined search string, literature searches will be run in English in at least five databases, three repositories and 10 websites, gathering both peer-reviewed and grey literature. Returns will be screened at title, abstract, and full text levels against defined inclusion criteria. Relevant metadata and effect data will be extracted from each study and used to write a narrative review and, where data allow, a meta-analysis of quantified effects. This review will provide a robust, up to date, consolidated and evaluated evidence base to inform future decision-making regarding the implementation of greening of grey infrastructure methods.

**Supplementary Information:**

The online version contains supplementary material available at 10.1186/s13750-026-00382-z.

## Background

Global coastlines are under increasing pressure from accelerating human population growth [44], rising sea levels [29] and the increasing intensity and frequency of extreme weather associated with a changing climate [36]. In the face of these changes, coastlines have been increasingly ‘hardened’ with coastal structures, such as seawalls, breakwaters, and groynes, to protect infrastructure and coastal populations. These structures now dominate urban coastlines [5, 10, 30]. Additionally, due to the growth in the blue economy and associated shifts to offshore renewable energy, there is an increase in the construction of offshore structures. This proliferation of coastal and offshore structures is causing the loss, modification and fragmentation of natural habitats [6], impacting native biodiversity [42, 56, 58], ecosystem functioning and, as a result, the provision of ecosystem services [39].

**Fig. 1:**
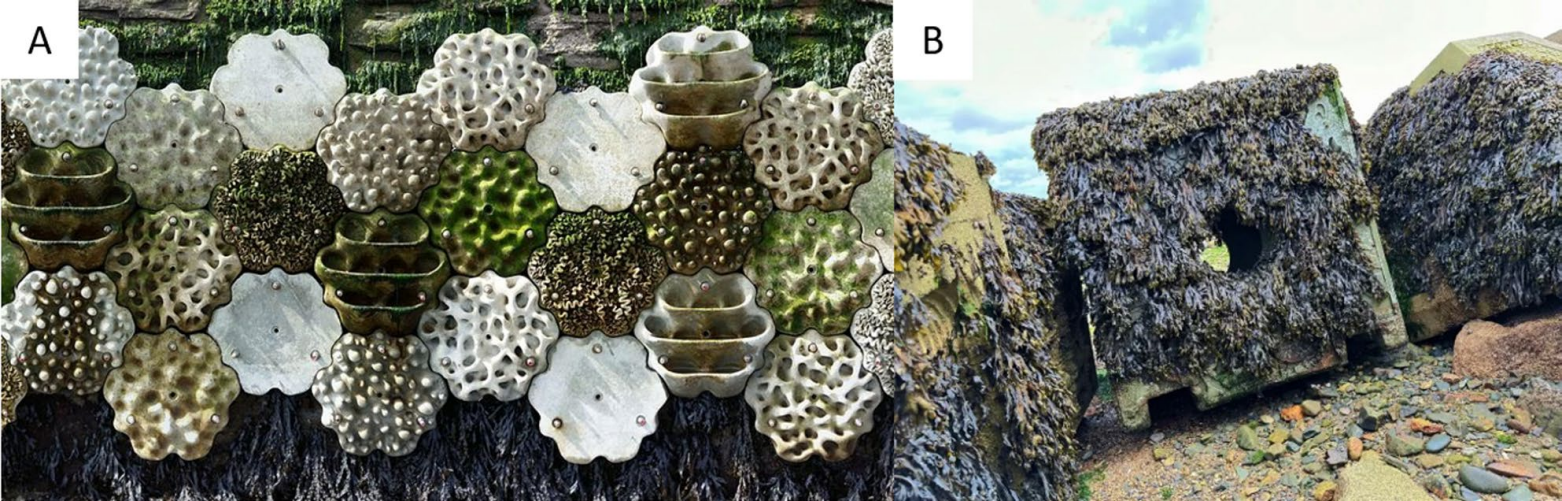
Examples of ‘Greening of Grey Infrastructure’ interventions, where conventional structures have been modified to provide an additional ecological function. **A** A seawall in Plymouth, UK, that has been enhanced using *Living Seawall* panels. Photo credit: Franz Bauer. **B** A rock armour block (*Reef Cube*) that has been designed with additional habitat features in Newlyn, UK. Photo credit: ARC Marine^®^

In an effort to combat the negative environmental impacts of coastal hardening and offshore infrastructure, ecological (eco-) engineering practices have been increasingly implemented worldwide [21, 55]. Eco-engineering applies ecological theory to traditional engineering practices to generate benefits for both humans and nature [21, 52]. The approach first gained recognition in urban freshwater and terrestrial habitats [28, 45], with examples including the construction of wetlands for urban wastewater treatment [50] and the implementation of green roofs to reduce water runoff and urban heat [14, 28]. Eco-engineering is now increasingly implemented in the marine environment, for example, by deploying artificial reefs to increase biodiversity and support tourism [2, 23, 61]. A specific type of eco-engineering aims to modify existing traditional grey infrastructure to enhance ecological value and support biodiversity. This approach, often referred to as ‘greening of grey infrastructure’ (GGI), and described using terms like ‘nature-inclusive design’ [35, 53] and ‘biophilic design’ [26, 37], is being applied to coastlines globally, particularly on coastal defence structures designed for non-ecological functions, such as seawalls or groynes [21, 41, 43]; Fig. 1. Increasingly, GGI is being considered for offshore structures, such as renewable energy infrastructure [17, 27, 48, 62] but applied case studies are still relatively rare. The aim of GGI is for structures to provide secondary environmental benefits, in addition to their primary engineered function (e.g., coastal protection, recreational access or energy production), thereby becoming multifunctional ‘ecologically enhanced structures’ [15, 40, 46].

Commonly, this ecological enhancement is achieved through the addition of topographic complexity onto otherwise featureless structures, increasing the microhabitat diversity [4, 22, 54]. The topographically complex features added to grey infrastructure commonly mimic those of natural rocky shores - the closest natural analogue to grey infrastructure - but in some instances, the added complexity has aimed to replicate habitat features of the specific shoreline habitats that have been displaced, such as oyster reefs and mangroves [59]. The range of different microhabitat types provided by rocky substrata offers refuge from predators, moisture retention, thermal buffering, and increased attachment space [4, 13, 16, 63]. An increase in surface topography has been shown to generally increase species diversity both in natural contexts [1, 3] and when manipulated through GGI modifications [19, 55].

One example of such GGI modification includes the creation of artificial rockpools by drilling pits into horizontal surfaces on existing breakwaters in the UK [18] and revetments in Malaysia [9], which led to significantly higher species richness compared to unaltered surfaces. Another widely-trialled modification involved the attachment of concrete habitat panels that mimic natural rocky shore features to vertical seawalls (an example shown in Fig. 1A), with a global study highlighting greater taxa richness on complex than on flat tiles at 10 out of 14 global locations [56]; see also [47]. These are examples of ‘retrofitting’ GGI interventions, where enhancements are made to pre-existing structures. These retrofitted enhancements currently dominate the field of GGI research. Ecological enhancements can, however, also be incorporated during initial construction (i.e., at the design stage prior to infrastructure deployment). For instance, the creation of complex boulders for placement in rock armour structures (Fig. 1B), or the omission of blocks in a newly-renovated seawall in Sydney, Australia [7, 8], and the manipulation of mortar in a newly built seawall in Devon, UK [24], both of which created crevices that supported higher taxa richness than external wall surfaces. While these targeted structural modifications (retrofitting and designing) show promise for ecological enhancement, their broader ecological effectiveness remains uncertain without systematic and long-term evaluation.

Greening of grey infrastructure is a rapidly increasing field [21], yet it remains a ‘young’ science not yet subjected to the critical evaluation necessary before being considered a mainstream solution. To date, studies have been largely conducted at small spatial and temporal scales, with low to no replication (but see [56]), despite it being recognised that the outcomes of ecologically enhanced marine structures vary across environmental contexts [11, 47, 55, 56]. As such, it is hard to draw generalised conclusions regarding the efficacy of specific GGI solutions. As GGI is becoming increasingly recognised as a potential contributor towards sustainable development, it is crucial that its implementation is justified and supported by robust scientific evidence. This is particularly important given the existing concerns over greenwashing strategies aimed at facilitating coastal development [19–21], with the risk of GGI making new developments more appealing, thus facilitating potentially damaging coastal development. Due to the multidisciplinary nature of GGI research, however, findings are likely dispersed across both the primary and grey literature, constituting a heterogeneous and fragmented evidence base spread across academics, practitioners, governments and regions. Two systematic reviews, undertaken by Strain et al. [55] and Evans et al. [19], have provided critical foundations for understanding the ecological outcomes of GGI interventions, describing evidence published prior to 2021. Since their publication, however, the volume and scope of research in this area has grown substantially. Moreover, only recently has GGI started to be applied at scale, and there is a need to assess whether results obtained from small-scale experiments also apply to scaled-up interventions [60]. There is therefore a need for an up-to-date, comprehensive synthesis of existing empirical research on the ecological effectiveness of GGI interventions, updating and building upon prior reviews [19, 55], to ensure that future applications are supported by sound scientific understanding.

To address the pressing need for an up-to-date evidence base, we propose to collate and synthesise in a systematic review, and where possible a meta-analysis, the best available global scientific evidence for the effectiveness of GGI interventions aimed at ecologically enhancing estuarine, coastal and offshore structures, using state-of-the-art evidence synthesis methodologies. The ecological effects of interest will include the abundance, biomass, composition and functional diversity of marine species on or immediately around ecologically enhanced structures. In this protocol, we present and detail the methodology that will be used to conduct the proposed systematic synthesis.

### Stakeholder and advisory group

This protocol was developed in consultation with an advisory group comprised of 16 relevant expert representatives from academia, industry, and government agencies. Consulted individuals specialised in ecological engineering research, coastal management, and civil engineering in the marine environment, with representatives from Europe, Oceania, North America and Africa, all working in different ecological, economic and social contexts. Experts were identified based on their demonstrated experience and contributions in the field of greening of grey infrastructure and ecological marine management. Selection criteria included relevant academic publications, applied project experience, or policy roles related to the greening of grey infrastructure. Individuals were contacted by email and invited to attend a video call. Advisors provided input into the research questions, aims and scope of the study, technical language included in the search string, and the identification of sources of grey literature. Members of the advisory group who agreed to be identified are listed in Additional File 1. Following substantial contribution, some of the advisors were invited to be co-authors on this protocol.

By synthesising the available evidence for the ecological effectiveness of GGI interventions, we aim to provide a robust, up-to-date evidence base to inform future decision-making. We anticipate this systematic review being of interest and use to a range of relevant stakeholders, particularly researchers, policy makers and practitioners, supporting the development of nature-inclusive designs and research practices, guiding policy development, and contributing to the sustainable management strategies for estuarine, coastal and offshore infrastructure.

### Objective of the review

#### Primary question

The primary objective of this systematic review is to answer the following question:

What are the effects of GGI interventions applied to marine structures on the diversity, abundance, biomass, composition, and functional diversity, of species on or around these ecologically enhanced structures?

The question is structured according to the PICO (Population, Intervention/Exposure, Comparator, Outcome) framework [51], with specific components outlined in Table 1, and a more comprehensive breakdown of the corresponding inclusion parameters provided below under ‘Eligibility Criteria’.

**Table 1:** PICO/PECO components of the primary question of the systematic review “What are the effects of GGI interventions applied to marine structures on the diversity, abundance, biomass, composition, and functional diversity, of species on or around these ecologically enhanced marine structures?”

PICO component	Included elements	Excluded elements
Population(s)	All macro marine species (Sessile and mobile organisms, including fish and macro-invertebrates, larger than 1 mm).	Microorganisms such as bacteria or viruses
Intervention(s)/Exposure(s)	All GGI interventions applied to marine structures i.e., addition, modification, or construction of ecological enhancements	Nature-based solutions that do not incorporate hard-engineered structures in some capacity (e.g., saltmarsh rehabilitation) Freshwater or terrestrial interventions Interventions involving grey infrastructure implemented or put at sea solely for an ecological purpose (e.g. artificial reefs without an independent engineering or functional purpose)
Comparator(s)	One or more of the following options: - Unmodified structure vs. ecologically enhanced structure (i.e., structure with intervention applied) - Natural environment vs. ecologically enhanced structure - Ecologically enhanced structure 1 (using one intervention) vs. ecologically enhanced structure 2 (using a different intervention) - Structure Before vs. After ecological enhancement A comparator (or ‘control’) is not essential for inclusion in the systematic review, but essential for inclusion in the subsequent meta-analysis	
Outcomes(s)	Abundance Biomass Species diversity Species composition Functional diversity	Non-biological outcomes Genetic or phylogenetic diversity Phenotypic diversity

The evidence base will include peer-reviewed publications, grey literature, and unpublished primary data, building on previous reviews [19, 55] and applying evidence synthesis methods from the Collaboration for Environmental Evidence [12]. Including grey literature is crucial for a balanced view of GGI interventions for two key reasons: (1) To minimise publication bias [57] and highlight ‘lessons learnt’, which may be important in a rapidly expanding field like GGI [21]; and (2) because many valuable GGI efforts are led by NGOs, consultants, agencies, and industry groups, producing non-academic reports that are often excluded from conventional reviews.

#### Secondary questions

Secondary objectives identified during discussions with advisors, are listed below in question form (Q1-7). Q 1–5 explore whether ecological outcomes vary with other factors (explanatory variables) that will be considered as potential modifiers in the proposed meta-analysis. Q 6–7 address broader, open-ended aspects of GGI interventions. Q7, less suited to quantitative synthesis, will be explored through narrative synthesis. Resource constraints, including time, available funding, and data availability, may limit the number of questions that can be addressed, nevertheless, a minimum of four will be aimed for.

- Q1. Does GGI intervention or enhancement type (defined in Additional File 1; Table 2) affect ecological outcomes (Additional File 1; Table 3)?
- Q2. Do ecological responses (Additional File 1; Table 3) to GGI interventions vary geographically (across classifications such as climatic zone, hemisphere, or ecoregion)?
- Q3. Do ecological responses (Additional File 1; Table 3) to GGI interventions increase with the spatial scale of interventions?
- Q4. Is there a difference in the ecological effects of structures retrofitted with habitat-enhancement features and those with built-in features?
- Q5. Does material type used in GGI interventions impact their ecological effectiveness?
- Q6. Do GGI interventions impact the presence of non-native species?
- Q7. What unintended consequences of GGI installations have been identified?

**Table 2:** Literature sources that will be systematically searched for relevant studies. Secondary sources will be searched if time allows

Priority sources to search	Secondary sources to search
Bibliographic databases: Web of Science Core Collection (Indexes: Science Citation Index Expanded (SCI-EXPANDED)--1970s-present Social Sciences Citation Index (SSCI)--1970-present Arts & Humanities Citation Index (AHCI)--1975-present Conference Proceedings Citation Index - Science (CPCI-S)--1990-present Conference Proceedings Citation Index - Social Science & Humanities (CPCI-SSH)--1990-present Emerging Sources Citation Index (ESCI)--2015-present )^a^ (https://www.webofscience.com/wos/woscc/smart-search) Scopus^a^ (https://www.scopus.com/home.uri) Dissertations and Thesis Database- ProQuest^a^ (https://www.proquest.com/index) Collaboration for Environmental Evidence library^b^ (https://environmentalevidence.org/completed-reviews/) Search Engines: Google Scholar^a^ (https://scholar.google.com/) Grey Literature Sources: FAO Document Repository^b^ (https://openknowledge.fao.org/home) Intergovernmental Panel on Climate Change (IPCC)^b^ (https://www.ipcc.ch/) International Union for Conservation of Nature (IUCN)^b^ (https://iucn.org/) UN Environment Programme (UNEP)^b^ (https://www.unep.org/)	Repositories: African Journals Online^b^ (https://www.ajol.info/index.php/ajol) Directory of Open Access Journals (DOAJ)^b^ (https://doaj.org/) Environmental Studies Program Information System (ESPIS)^b^ (https://coast.noaa.gov/digitalcoast/tools/espis.html) ResearchGate^a^ (https://www.researchgate.net/search) Grey Literature Sources: Archives Africa^b^ (https://archives-africa.org/) Department for Environment, Food and Rural Affairs (DEFRA)^b^ (https://www.gov.uk/government/organisations/department-for-environment-food-rural-affairs) Department of Forestry, Fisheries and the Environment (DFFE), South Africa^b^ (https://www.dffe.gov.za/) British Ecological Society (BES)^b^ (https://www.britishecologicalsociety.org/) Centre for Environment, Fisheries and Aquaculture Science (Cefas publication hub)^b^ (https://www.cefas.co.uk/) CSIR ResearchSpace (Council for Scientific and Industrial Research, South Africa)^b^ (https://www.csir.co.za/) Department of Fisheries and Oceans Canada^b^ (https://www.dfo-mpo.gc.ca/index-eng.html) Environment Agency (EA)^b^ (https://www.gov.uk/government/organisations/environment-agency) European Environment Agency (EEA)^b^ (https://www.eea.europa.eu/en) Korean Society of Fisheries and Aquatic Sciences^b^ (https://www.kosfas.or.kr/eng/) Marine Conservation Alliance^b^ (https://marineconservationalliance.org/) Marine Management Organisation (MMO)^b^ (https://www.gov.uk/government/organisations/marine-management-organisation) Marine Scotland^b^ (https://marine.gov.scot/) National Environmental Research Council^b^ (https://www.ukri.org/councils/nerc/) National Institute of Water and Atmospheric Research^b^ (https://niwa.co.nz/) National Oceanic and Atmospheric Administration (NOAA)^b^ (https://www.noaa.gov/) Natural England (NE)^b^ (https://www.gov.uk/government/organisations/natural-england) Natural Resources Wales (NRW)^b^ (https://naturalresources.wales/?lang=en) Northern Ireland Environmental Agency^b^ (https://www.daera-ni.gov.uk/articles/northern-ireland-environment-agency) Royal Netherlands Institute of Sea Research^b^ (https://www.nioz.nl/en) Scottish Association for Marine Science (SAMS)^b^ (https://www.sams.ac.uk/) Scottish Environmental Protection Agency^b^ (https://www.sepa.org.uk/) Western Indian Ocean Marine Science Association (WIOMSA)^b^ (https://www.wiomsa.org/)

^a^Sources that will be searched using agreed search string (or variant thereof), ^b^Sources where search string cannot be used and manual searches will be performed

## Methods

This review will follow the Collaboration for Environmental Evidence guidelines (Collaboration for Environmental Evidence, [12]). This protocol conforms to the ROSES (Reporting standards for Systematic Evidence Syntheses) standards ([32]; see Additional File 2).

### Searching for articles

A search string was developed using a scoping exercise conducted in the Web of Science Core Collection database (using the University of Plymouth subscription). Scoping searches were undertaken to explore the efficacy of a variety of search terms, building on search strings used in previous eco-engineering systematic reviews undertaken by Strain et al. [55] and Evans et al. [19]. The evolution of the search string is presented in Additional File 3. The final search string, presented below, produced the highest efficiency, with 3,778 hits as of 24/07/2025 on Web of Science, retrieving 28/30 benchmark publications, with the two publications not included being unavailable on Web of Science. The search string comprises Intervention, Structure, Environment, and Outcome qualifying terms. We also specified excluded terms that led to irrelevant results. Population terms were not stipulated because studies investigating all macro marine species will be included. Comparator terms were also not included because, for the systematic review, we deem it non-essential for a comparator to be included in usable studies. Studies included in the subsequent meta-analysis will be selected based on data availability and will be required to have a comparator.

#### Search languages

The searches will be conducted in English only due to resource constraints.

#### Search strings

##### Intervention qualifying terms

TS= (bioblock$ OR “bio* enhanc*” OR “bio* mimic*” OR biophilic OR complex* OR “eco-eng*” OR “ecoeng*” OR “eco* engineer*” OR “eco* enhance*” OR “eco-design*” OR “eco-innovation$” OR “eco-restor*” OR “eco-sensitive” OR “eco-tech*” OR “engineer* complex*” OR GGI OR GBGI OR “green eng*” OR greening NEAR/3 grey OR “habitat enhanc*” OR “habitat augment*” OR “habitat enhanc* panel$” OR “habitat modif*” OR “habitat panel$” OR “habitat tile$” OR heterogen* OR IGGI OR install* OR “intertidal mitigat*” OR “living shore*” OR “living seawall$” OR manipulat* OR microhabitat$ OR microtopograph* OR mimic* OR “nature-based solution$” OR “nature-inclu* design*” OR NbS OR rough* OR “structur* complex*” OR “substrat* enhanc*” OR textur* OR topograph* OR transplant*).

AND

##### Artificial structure qualifying terms

TS= (armor* OR armour* OR “anthropogenic structure$” OR “anthropogenic infrastructure” OR “artificial habitat$” OR “artificial infrastructur*” OR “artificial shore*” OR “artificial structur*” OR breakwater$ OR “built structure$” OR bulkhead$ OR caisson$ OR “coast* defen*” OR “coast* hard*” OR “coast* protect*” OR embankment$ OR “engineer* structur*” OR “coast* guard*” OR dike$ OR dock$ OR dolphin$ OR dyke$ OR “float* dock*” OR “float* structure*” OR “flood* defenc*” OR “grey infrastructure” OR “gray infrastructure” OR groin$ OR groyne$ OR humanmade OR “human-made” OR infrastructure OR jett* OR “manmade structure$” OR “man-made structure$” OR marina$ OR “marine construction$” OR “marine urban*” OR mooring$ OR “ocean sprawl” OR “offshore structur*” OR “rig$” OR pier$ OR piling$ OR pontoon$ OR port$ OR quay OR revetment$ OR riprap OR “sea defence$” OR seawall* OR “sea wall*” OR “shore* protect*” OR “storm surge barrier$” OR “tidal energy” OR tile$ OR “underwater structure$” OR “wave break*” OR wavescreen$ OR “wind turbine$” OR “oil and gas” OR “oil and gaz” OR “oil & gas” OR “oil & gaz” OR “petroleum installation*” OR “windfarm$” OR “wind farm$” OR MREI OR “marine renewable$” OR “wave farm*” OR “tidal energy” OR “tidal stream*”).

AND.

##### Environment terms

TS= (coast* OR estuar* OR intertidal OR marine OR ocean* OR offshore OR subtidal OR shore* OR “water column” OR pelagic OR benthic).

AND.

##### Outcome qualifying terms

TS= (assemblage$ OR diversit* OR abundance$ OR biodiversit* OR biomass* OR “community structure$” OR “community composition$” OR “functional composition$” OR “functional evenness” OR “functional group$” OR “functional richness” OR “functional trait composition” OR “species composition” OR “species evenness” OR “species presence” OR “species richness” OR “species similarit*” OR “taxonomic composition$” OR “taxonomic richness”).

##### Excluded terms

AND NOT.

TS= (oceanography OR “zostera marina” OR “bottlenose dolphin$”).

The search string above uses Web of Science syntax (more information in Additional File 3). To improve search precision, NOT operators were used selectively to remove highly specific terms that altered the intended meaning of included keywords and retrieved large numbers of irrelevant literature. Trial searches and title screening confirmed that no relevant records were excluded.

#### Sources of literature to be searched

Using the search string detailed above, we will search a wide variety of literature sources (Table 2) to maximise retrieved data. The search string will be amended in accordance with the requirements of each literature source, including the alteration of syntax (see Additional File 3). Grey literature sources that do not accept search strings will be searched manually using keywords and basic syntax (listed in Additional File 3).

All searches will be undertaken from a single laptop to ensure that all settings are consistent, and a record of all searches will be kept. All hits will be considered while searching bibliographic databases. When using search engines, such as Google Scholar, the first 200 returns will be considered as recommended by Haddaway et al., [31]. Due to time constraints, literature sources have been split into priority sources and secondary sources (Table 2). Searches, screening and data extraction will be undertaken first on studies retrieved from priority sources, followed, if time allows, by studies returned from secondary sources. Large bibliographic databases, or grey literature sources with global scope are defined as primary sources. Secondary sources include repositories requiring manual searching, and location-specific grey literature sources.

#### Additional searches

Snowballing of cited references will be applied to systematic reviews and meta-analyses that pass the screening. Specifically, all references cited in existing systematic reviews by Strain et al., [55] and Evans et al., [19] will be collated to ensure their inclusion in the screening process, directly building on and extending the scope of earlier efforts. Additional literature and unpublished primary data will be obtained directly from the Advisory Group members and from external stakeholders identified from existing ecological engineering networks (e.g., The Marine Eco-Engineering UK Working Group). Grey literature and unpublished research will also be requested through social media calls.

#### Assembling a library of search results

Publications will be compiled in the reference manager Zotero and subsequently uploaded into the review management software Covidence where they will be deduplicated using advanced artificial intelligence (AI) processes.

#### Comprehensiveness of the searches

A list of 31 benchmark publications was compiled prior to search string testing by members of the review team and advisory group based on their knowledge of the research area (see Additional File 1; Table 1). The list of benchmark publications is dominated by intertidal studies which reflects the current evidence base. The search string was refined until > 90% of all benchmark publications were retrieved.

### Article screening and study eligibility criteria

#### Screening process

Publications will be screened in two stages: (1) title and abstract level; (2) full-text level. At each stage, the decision will be made whether to exclude or include each publication in accordance with the eligibility criteria (defined below). If a decision is unclear at first stage, publications will be included and a final decision will be made at full-text read. Publications excluded at full-text stage will be assigned the reason for their exclusion (see Additional File 1; Table 4) and provided as part of the systematic review report. When publications cannot be accessed in full using the University of Plymouth’s subscriptions, contact with authors will be attempted to request full-text publications. Any publications excluded due to unavailability will be recorded.

Screening will involve three independent reviewers. If not already trained in evidence synthesis and article screening, reviewers will undertake appropriate training using Collaboration for Environmental Evidence (CEE) resources. Any publications authored or co-authored by any members of the review team will be screened by an impartial reviewer. Fleiss’s Kappa test [25] will be used to assess consistency between reviewers. A random subset of 100 publications will be screened independently by each reviewer, substantial (K = 0.61–0.8) or almost perfect (K = 0.81-1.0) agreement will warrant discussions about disagreements but not require further training. A Kappa score of less than 0.61 will elicit further training in the form of refreshment of the inclusion and exclusion criteria, joint discussion of example studies, and a further Fleiss’ Kappa test for 50 additional publications. This process will repeat until a score of above 0.61 is reached.

#### Eligibility criteria

Publications meeting the following criteria will be included in the review, according to the relevant PICO components described above (Table 1).

#### Eligible populations

All macro marine species on and around ecologically enhanced marine structures will be considered. Macro marine species will include both sessile and mobile organisms, including fish and macro-invertebrates, larger than 1 mm (visible to the naked eye). For the purpose of this review, ecologically enhanced marine structures are defined as artificial estuarine and fully marine structures (including both coastal and offshore structures) that serve a non-environmental primary function but that have been constructed or modified with the addition of habitat-enhancing features (having been subject to a GGI ‘Intervention’ described below). The geographical scope will be global.

#### Eligible interventions

All GGI interventions (i.e., eco-engineering intervention applied to a relevant structure with the aim of providing secondary environmental benefits) will be considered. These include (but are not limited to): the addition of texture, protrusions or other topographic features to structures; the addition of habitat-forming taxa to structures; the modification of structures through processes such as drilling. Interventions may have been performed at either the design/building stage (pre-construction) or retrospectively through retrofitting. A preliminary list of known interventions can be found in Additional File 1. Other relevant intervention types encountered during literature searches will be added to the list on an ad hoc basis. Structures designed solely for ecological purposes, and those in freshwater environments, will be excluded as well as nature-based solutions that do not incorporate hard-engineered structures in some capacity (e.g., saltmarsh rehabilitation).

#### Eligible comparators

Suitable comparisons are not always possible in the marine environment, therefore, comparators are not essential for the inclusion of studies into the narrative synthesis. Studies will however, be required to include at least one comparator for inclusion in any subsequent meta-analysis, using one or more of the following designs:

- Unmodified structure vs. ecologically enhanced structure (i.e., structure with GGI intervention applied).
- Natural environment vs. ecologically enhanced structure.
- Ecologically enhanced structure 1 (using one intervention) vs. ecologically enhanced structure 2 (using a different intervention).
- Structure Before vs. After ecological enhancement.

#### Eligible outcomes

Relevant outcomes include those pertaining to abundance, biomass, species diversity, species composition, or functional abundance and diversity of macro marine species (defined above) on and immediately around ecologically enhanced marine structures (see list of possible metrics in Additional File 1, Table 3; others may be encountered during searches and will be added on an ad hoc basis). We will not be including studies on genetic or phylogenetic diversity, phenotypic diversity, or diversity related to micro-organisms such as bacteria and viruses.

#### Eligible types of study design

Only in situ experimental and observational studies will be included. Systematic reviews and meta-analyses will be included only if they provide new data. Laboratory studies, modelling studies and traditional narrative reviews will be excluded. In this review, a ‘study’ is defined as a distinct experimental or comparative unit. Therefore, if a single publication reports data collected from multiple locations or a variety of interventions, each of these will be treated as an independent study in the analysis. If data is presented collectively for multiple studies, authors will be contacted to request the separated data.

### Study validity assessment

Studies that meet eligibility criteria will undergo critical appraisal to assess validity using the CEE critical appraisal tool (presented in Additional File 4). This tool has been designed by the Collaboration for Environmental Evidence to effectively evaluate the risk of bias of primary studies assessing effectiveness of interventions in environmental management [38]. Studies that suffer from reporting bias (‘unclear’ validity, e.g. due to missing key methodological information about study design or intervention or if no suitable comparator is provided) will be excluded from quantitative synthesis. All studies excluded after critical appraisal will be listed in the review, together with a reason for exclusion. Using the CEE tool, all other studies will be allocated low, medium, or high risks of bias which will be used in sensitivity analysis during quantitative synthesis (as defined in ‘meta-analysis’ section below). All critical appraisals will be undertaken by one reviewer, but 10% of studies will be critically appraised by a second reviewer to ensure agreement. No reviewer will critically appraise their own publication.

### Data coding and extraction strategy

For each study that passes the second screening stage, the following associated meta-data and effect data will be coded in a standardised framework presented as an Excel database (see Additional File 5). Coded information (meta-data) will include: (1) A unique identifier for each study; (2) Bibliographic details (authors, year of publication, title, search source); (3) Information related to the study (including but not limited to, study year, study duration, study site, study design); (4) PICO components (as defined in Table 1); (5) Detailed geographic information (including climatic zone, hemisphere, ecoregion); and (6) Additional meta-data [where available, including but not limited to, time elapsed since intervention, structure type (e.g., seawall, groyne, pontoon), surrounding habitat type, size of intervention area (m^2^), presence of non-native species, unintended outcomes]. A full working framework is available in Additional File 5. For each study, detailed information of the reported outcomes will be included, as either descriptive text (see below ‘narrative synthesis’ section) and/or when available, quantified effect (see below ‘meta-analysis’ section). Extracted data records will be made available alongside the final publication as additional files.

Data from 10 randomly selected publications that passed title/abstract screening will be extracted independently by two reviewers. Kappa scores will be calculated to test agreement between reviewers on data and selected meta-data. As above, if the Kappa score is less than 0.61, then another 10 publications will be coded and compared again following discussions. Once substantial agreement is reached, the remaining data extraction process will be carried out by one reviewer only per paper, splitting the publications across reviewers. Publication authors will be contacted when data are missing.

#### Data extraction for meta-analysis

For each study included in the systematic review and containing relevant quantified effect data, the sample sizes, means, and measures of variance (e.g., confidence intervals, standard errors, or standard deviations) of the relevant metrics (above) will be recorded for both the intervention and the control site(s). If not provided in the publication, summary statistics will be generated from raw data where possible. Publication authors will be contacted when data are missing. GetData graph digitizer will be used to extract data when presented in figures.

### Potential effect modifiers

Based on the expertise of the advisory group and review team, and in line with the review questions (see ‘secondary questions’ above), the following potential effect modifiers will be coded and considered:

- GGI intervention or enhancement type (defined in Additional File 1; Table 2).
- Geographical variation (classifications such as climatic zone, hemisphere and ecoregion).
- Spatial scale of intervention.
- Material type.

This is not an exhaustive list but the core focus of the systematic review. A full list of coded information can be found in Additional File 5.

### Data synthesis and presentation

Findings of this systematic review will be presented in a narrative synthesis, and, where data allow, in a quantitative analysis (meta-analysis).

#### Narrative synthesis

In the first instance, the nature and distribution of the evidence identified will be summarised. Metadata will be presented descriptively, grouping studies according to intervention type, structure type, geographic location, and other relevant groupings, and descriptive statistics, tables, figures and narrative descriptions used to describe the evidence base. We will identify evidence clusters, where research has been heavily focused on specific interventions, species or locations, as well as areas where there may be research gaps. Developments in the body of literature over time will also be examined, with particular attention given to changes since the publication of the previous systematic reviews by Strain et al., [55], and Evans et al., [19], to contextualise new findings and assess emerging trends and shifts in research focus.

Findings from studies will be presented narratively, structured around the review’s primary and secondary questions, focusing on key themes such as ecological outcomes associated with different greening interventions, geographic distribution and spatial scale, the presence of non-native species, the differences between retrofitted and newly built GGI structures and the influence of material types. Additionally, unintended outcomes of GGI will be discussed narratively regardless of the possibility of quantitative analysis. Descriptive text will also include a description of measured outcomes and the reported direction of effect at the individual study level (e.g. positive, negative, mixed, or no detectable effect), alongside key study characteristics. Direction of effect will be used solely to descriptively summarise and contextualise individual study findings and to aid interpretation of heterogeneity across studies. No vote-counting or tallying of studies based on directions of effect will be undertaken. Findings will be organised according to intervention type, structure type and other relevant study characteristics to allow for meaningful comparisons across studies.

#### Meta-analysis

If sufficient empirical data are available, quantitative analysis will take place in the form of a meta-analysis. Comparable effect sizes of eligible studies will be calculated using the most appropriate metric for the data type, such as the natural log transformed response ratio (lnRR) [34] or Hedges’ *g* [33, 34]. Additionally, sensitivity analysis will be conducted to evaluate the potential impact of the inclusion of low validity studies, and publication bias will be investigated using funnel plots and related statistical methods [49]. Sensitivity analysis will involve the comparisons of two sets of results, the first being the results from analyses where all studies are included, and the second being the results from analyses that includes only studies with medium or high validity, with the exclusion of low validity studies. Comparisons between the two sets of results will allow us to assess how sensitive the findings are to the inclusion of low-validity studies. Quantitative outcomes will be graphically presented. Meta-analysis will be conducted in R v4.2.0. (R Core Team, 2022), specific methods will depend on effect sizes and model types.

## Supplementary Information

Below is the link to the electronic supplementary material.

Supplementary Material 1.

Supplementary Material 2.

Supplementary Material 3.

Supplementary Material 4.

Supplementary Material 5.

## Data Availability

All data generated or analysed during this study are included in this published article and its supplementary information files.
