# Supplementary material for: Global evidence for the ecological effects of greening of grey infrastructure: a systematic review protocol
Source: Environ Evid. 2026 Mar 5;15:3. doi: 10.1186/s13750-026-00382-z (PMC13072617; doi:10.1186/s13750-026-00382-z)
Supplement: Supplementary file 1 — Supplementary Material 1. [file 13750_2026_382_MOESM1_ESM.docx]

Supplementary Information 1

**List of Advisory Group Members**

Dr Melanie Bishop* - Macquarie University, Australia

Mr Tom Birbeck- ARC Marine, UK

Mr Austin Brown- ARUP, UK

Mr Jon Challis- Inland and Coastal Marina Systems, UK

Dr Katherine Dafforn*- University of Massachusetts Boston, United States

Dr Ferrante Grasselli*- ARC Marine, UK

Mr Samuel Hickling- ARC Marine, UK

Dr Mariana Mayer-Pinto*- University of New South Wales, Australia

Dr Kathryn O’Shaughnessy*- Dauphin Island Sea Lab, USA

Dr Francesca Porri*- South African Institute for Aquatic Biodiversity and Rhodes University, South Africa

Mr Justin Ridgewell- Environment Agency and Cornwall Council, UK

Dr Rebecca Smith*- University of Cambridge, UK

Mr Oliver Shortall- Inland and Coastal Marina Systems, UK

Dr Elisabeth Strain*- University of Tasmania, Australia

Ms Amelia Sturgeon- Plymouth City Council and Tamar Estuaries Consultative Forum, UK

Ms Sara Wordley - Marine Management Organisation, UK

*denotes advisors who were invited to become co-authors due to their significant contribution

**Table 1. List of benchmark publications for inclusion in the systematic review**

| **Lead Author(s)** | **Year** | **Title** | **Journal Name** | **DOI** |
| --- | --- | --- | --- | --- |
| Bishop | 2022 | Complexity–biodiversity relationships on marine urban structures: reintroducing habitat heterogeneity through eco-engineering | Philosophical Transactions of the Royal Society B | <https://doi.org/10.1098/rstb.2021.0393> |
| Bone | 2022 | Estuarine Infauna Within Incidentally Retained Sediment in Artificial Rockpools | Frontiers in Marine Science | <https://doi.org/10.3389/fmars.2021.780720> |
| Bradford | 2020 | Provision of refugia and seeding with native bivalves can enhance biodiversity on vertical seawalls | Marine Pollution Bulletin | <https://doi.org/10.1016/j.marpolbul.2020.111578> |
| Cacabelos | 2016 | Material type and roughness influence structure of inter-tidal communities on coastal defences | Marine Ecology | <https://doi.org/10.1111/maec.12354> |
| Cacabelos | 2018 | Patchiness in habitat distribution can enhance biological diversity of coastal engineering structures | Aquatic Conservation: Marine and Freshwater Ecosystems | <https://doi.org/10.1002/aqc.2972> |
| Chapman | 2009 | Engineering novel habitats on urban infrastructure to increase intertidal biodiversity | Oecologia | <https://doi.org/10.1007/s00442-009-1393-y> |
| Chapman | 2011 | Evaluation of ecological engineering of “armoured” shorelines to improve their value as habitat | Journal of Experimental Marine Biology and Ecology | <https://doi.org/10.1016/j.jembe.2011.02.025> |
| Chee | 2020 | Drill-Cored Artificial Rock Pools Can Promote Biodiversity and Enhance Community Structure on Coastal Rock Revetments at Reclaimed Coastlines of Penang, Malaysia | Tropical Conservation Science | <https://doi.org/10.1177/1940082920951912> |
| Clynick | 2007 | Effects of epibiota on assemblages of fish associated with urban structures | Marine Ecology Progress Series | <https://doi.org/10.3354/meps332201> |
| Dugan | 2011 | Estuarine and Coastal Structures: Environmental Effects, A Focus on Shore and Nearshore Structures | Treatise on Estuarine and Coastal Science | <https://doi.org/10.1016/B978-0-12-374711-2.00802-0> |
| Evans | 2015 | Drill-cored rock pools: an effective method of ecological enhancement on artificial structures | Marine and Freshwater Research | <https://doi.org/10.1071/MF14244> |
| Evans | 2021 | Replicating natural topography on marine artificial structures – A novel approach to eco-engineering | Ecological Engineering | <https://doi.org/10.1016/j.ecoleng.2020.106144> |
| Firth | 2014 | Between a rock and a hard place: Environmental and engineering considerations when designing coastal defence structures | Coastal Engineering | <https://doi.org/10.1016/j.coastaleng.2013.10.015> |
| Hair and Bell | 1992 | Effects of Enhancing Pontoons on Abundance of Fish: Initial Experiments in Estuaries | Bulletin of Marine Science | <https://www.researchgate.net/publication/233707761_Effects_of_Enhancing_Pontoons_on_Abundance_of_Fish_Initial_Experiments_in_Estuaries> |
| Hall | 2018 | Ecological enhancement techniques to improve habitat heterogeneity on coastal defence structures | Estuarine, Coastal and Shelf Science | <https://doi.org/10.1016/j.ecss.2018.05.025> |
| Jackson | 2015 | The influence of engineering design considerations on species recruitment and succession on coastal defence structures | Thesis | https://researchportal.plymouth.ac.uk/en/studentTheses/the-influence-of-engineering-design-considerations-on-species-rec |
| Kingma | 2024 | Guardians of the seabed: Nature-inclusive design of scour protection in offshore wind farms enhances benthic diversity | Journal of Sea Research | <https://doi.org/10.1016/j.seares.2024.102502>. |
| Langhamer | 2012 | Artificial Reef Effect in relation to Offshore Renewable Energy Conversion: State of the Art | Scientific World Journal | <https://doi.org/10.1100/2012/386713> |
| Liversage | 2017 | Availability of microhabitats explains a  widespread pattern and informs theory on ecological engineering of boulder reefs. | Journal of Experimental Marine Biology and Ecology | <https://doi.org/10.1016/j.jembe.2017.01.013> |
| Loke | 2017 | The effects of manipulating microhabitat size and variability on tropical seawall biodiversity: field and flume experiments | Journal of Experimental Marine Biology and Ecology | <https://doi.org/10.1016/j.jembe.2017.01.024> |
| Loke and Todd | 2016 | Structural complexity and component type increase intertidal biodiversity independently of area | Ecology | <https://doi.org/10.1890/15-0257.1> |
| MacArthur | 2019 | Maximising the ecological value of hard coastal structures using textured formliners | Ecological Engineering | <https://doi.org/10.1016/j.ecoena.2019.100002> |
| Martins | 2010 | Enhancing stocks of the exploited limpet *Patella candei* d’Orbigny via modifications in coastal engineering | Biological Conservation | <https://doi.org/10.1016/j.biocon.2009.10.004> |
| Morris | 2017 | Increasing habitat complexity on seawalls: Investigating large- and small-scale effects on fish assemblages | Ecology and Evolution | <https://doi.org/10.1002/ece3.3475> |
| Paalvast | 2012 | Pole and pontoon hulas: An effective way of ecological engineering to increase productivity and biodiversity in the hard-substrate environment of the port of Rotterdam | Ecological Engineering | <https://doi.org/10.1016/j.ecoleng.2012.04.002> |
| Pardo | 2023 | A synthesis review of nature positive approaches and coexistence in the offshore wind industry | ICES Journal of Marine Science | <https://doi.org/10.1093/icesjms/fsad191> |
| Perkol-Finkel | 2018 | Seascape architecture – incorporating ecological considerations in design of coastal and marine infrastructure | Ecological Engineering | <https://doi.org/10.1016/j.ecoleng.2017.06.051> |
| Sella and Perkol-Finkel | 2015 | Blue is the new green – Ecological enhancement of concrete based coastal and marine infrastructure | Ecological Engineering | <https://doi.org/10.1016/j.ecoleng.2015.09.016> |
| Strain | 2020 | A global analysis of complexity–biodiversity relationships on marine artificial structures | Global Ecology and Biogeography | <https://doi.org/10.1111/geb.13202> |
| Strain | 2020 | Interacting effects of habitat structure and seeding with oysters on the intertidal biodiversity of seawalls | PLOS ONE | <https://doi.org/10.1371/journal.pone.0230807> |
| Taira | 2020 | Ecological engineering across organismal scales: trophic-mediated positive effects of microhabitat enhancement on fishes | Marine Ecology Progress Series | https://doi.org/10.3354/meps13462 |

**Table 2. Categories of greening of grey interventions. Categories informed by reviews undertaken by Strain et al. (2018) and Evans et al. (2021) and information from the advisory group. Table modified from Evans et al. (2021)**

| **Intervention Type** | **Definition and Scale** | **Comments on definition** |
| --- | --- | --- |
| Textures surfaces (<1 mm) | Micro-scale roughness applied to entire surface that produces depressions and/or elevations ≤ 1mm | Defined by Strain et al. (2018) |
| Natural rocky reef topography | The full fingerprint of substrate topography found in natural rocky habitats. | Defined by Evans et al. (2021) |
| Groove habitats (1-50mm) | Depressions with a length to width ratio >3:1 and depth 1–50 mm | Defined by Evans et al. (2021) |
| Crevice habitat (>50mm) | Depressions with a length to width ratio >3:1 and depth >50 mm | Defined by Evans et al. (2021) |
| Pit habitats (1-50mm) | Depressions with a length to width ratio ≤3:1 and depth 1–50 mm | Defined by Evans et al. (2021) |
| Hole habitats (>50mm) | Depressions with a length to width ratio ≤3:1 and depth >50 mm that do not retain water during low tide in intertidal environments | Defined by Evans et al. (2021) |
| ‘Rock pools’ (>50mm) | Depressions with a length to width ratio ≤3:1 and depth ≥50 mm that retain water during low tide in intertidal environments | Defined by Evans et al. (2021) |
| Small protrusions (1-50mm) | Elevations with a length to width ratio ≤3:1 that protrude 1–50 mm from the substratum | Defined by Evans et al. (2021) |
| Small ridges or ledges (1-50mm) | Elevations with a length to width ratio >3:1 that protrude 1–50 mm from the substratum | Defined by Evans et al. (2021) |
| Large protrusions (>50mm) | Elevations with a length to width ratio ≤3:1 that protrude >50 mm from the substratum | Defined by Evans et al. (2021) |
| Large ridges or ledges (>50mm) | Elevations with a length to width ratio >3:1 that protrude >50 mm from the substratum | Defined by Evans et al. (2021) |
| Short flexible habitats (1-50mm) | Flexible protruding materials such as rope, ribbon or twine 1–50 mm in length | Defined by Evans et al. (2021) |
| Long flexible habitats (>50mm) | Flexible protruding materials such as rope, ribbon or twine >50 mm in length | Defined by Evans et al. (2021) |
| Transplant or seed organisms onto artificial structures | Actions taken to attach live organisms at any life stage onto structures, with the aim of generating self-sustaining populations | Defined by Evans et al. (2021) |
| Small adjoining cavities or ‘swimthrough’ habitats (<100m) | Adjoining internal cavities sheltered from, but with access to/from, outside the structure. Dimensions can vary but are ≤100 mm in any direction | Defined by Evans et al. (2021) |
| Large adjoining cavities or ‘swimthrough’ habitats (>100mm) | Adjoining internal cavities sheltered from, but with access to/from, outside the structure. Dimensions can vary but are >100 mm in any direction | Defined by Evans et al. (2021) |
| Groves *and* small protrusions, ridges or ledges (1-50mm) | ‘Groove habitats’ are depressions with a length to width ratio >3:1 and depth 1–50 mm. ‘Small protrusions’ are elevations with a length to width ratio ≤3:1 that protrude 1–50 mm from the substratum. ‘Small ridges and ledges’ are elevations with a length to width ratio >3:1 that protrude 1–50 mm from the substratum. | Defined by Evans et al. (2021) |
| Reduce slope | Actions taken to reduce the inclination of structures without increasing the footprint, with the aim of enhancing their biodiversity. | Defined by Evans et al. (2021) |
| Light penetrating surfaces | The modification of structures to allow more sunlight to reach the water below, using the inclusion of glass (or other transparent material), in surrounding structures. | Defined by authors. |
| Combination of intervention types | A combination of any of the above intervention types on a single structure | Defined by authors. |

**Table 3. List of possible abundance, biomass, species diversity, species composition and functional diversity metrics for inclusion in the systematic review**

| **Category** | **Metric** |
| --- | --- |
| **Abundance and/or Biomass** | Percentage Cover |
|  | Total Biomass |
|  | Individual Counts |
| **Species Diversity** | Species Richness (S) |
|  | Shannon Index (H’) |
|  | Simpson’s Index (D) |
|  | Inverse Simpson’s Index (1/D) |
|  | Pielou’s Evenness Index |
|  | Berger-Parker Index |
| **Species Composition** | Jaccard Index |
|  | Jaccard Beta Diversity |
|  | Bray Curtis Dissimilarity |
|  | Bray-Curtis Beta Diversity |
|  | Sørensen Index (Bray-Curtis Similarity) |
|  | Sørensen Beta Diversity |
| **Functional Diversity** | Functional Richness (FRic) |
|  | Functional Evenness (FEve) |
|  | Functional Divergence (FDiv) |
|  | Functional Dispersion (FDis) |
|  | Community-Weighted Mean (CWM) |
|  | Rao’s Quadratic Entropy |
|  | Functional Redundancy (FR) |
|  | Functional Beta Diversity |
|  | Functional Trait Composition Analysis |

**Table 4. Possible reasons for exclusion at the full text screening stage, additional reasons for exclusion may be added to the list on an ad hoc basis**

| **Reason for Exclusion** | **Example** |
| --- | --- |
| Irrelevant Population | Microorganisms such as bacteria or viruses |
| Irrelevant Intervention | Nature-based solutions that do not incorporate hard-engineered structures in some capacity (e.g. saltmarsh rehabilitation), freshwater or terrestrial interventions, interventions without a function separate from ecological enhancement |
| Irrelevant Outcomes | Non-biological outcomes, genetic or phylogenetic diversity, phenotypic diversity |
| Irrelevant Study Design | Laboratory studies, modelling studies, systematic reviews and meta-analyses that do not provide new data, traditional narrative reviews |
