# Supplementary material for: Global evidence for the ecological effects of greening of grey infrastructure: a systematic review protocol
Source: Environ Evid. 2026 Mar 5;15:3. doi: 10.1186/s13750-026-00382-z (PMC13072617; doi:10.1186/s13750-026-00382-z)
Supplement: Supplementary file 3 — Supplementary Material 3. [file 13750_2026_382_MOESM3_ESM.pdf]

Item number	Section / sub-section	Topic	Description	Further explanation	Checklist/Meta-data	Author response	Comments
1	Title	Title	The title must indicate that it is a systematic review protocol, and must indicate if it is an update/amendment: e.g. "A systematic review update protocol...".	The title should normally be the same or very similar to the review question.	Meta-data	Global evidence for the ecological effects of greening of grey infrastructure: A systematic review protocol	
2	Type of review	Type of review	Select one of the following types of review: systematic review, systematic review update, systematic review amendment, systematic review from a systematic map	See CEE Guidance on amendments and updates [1]	Meta-data	systematic review	
3	Authors contacts	Authors contacts	The full names, institutional addresses, and email addresses for all authors must be provided.		Checklist	Yes	
4	Abstract	Structured summary	Abstract must not exceed 350 words and must include two sections 1) Background, the context and purpose of the review, including the review question; 2) Methods, how the review will be conducted and the outputs that are expected (specifically mention search strategy, inclusion criteria, critical appraisal, data extraction and synthesis).		Checklist	Yes	
5	Background	Background	Describe the rationale for the review in the context of what is already known. Protocol must indicate why this study was necessary and what it aims to contribute to the field.	A theory of change and/or conceptual model can be presented that links the intervention or exposure to the outcome.	Checklist	Yes	
6	Stakeholder engagement	Stakeholder engagement	The planned/actual role of stakeholders throughout the review process (e.g. in the formulation of the question) must be described and explained (using a broad definition of 'stakeholder', including e.g. researchers, funders and other decision-makers; see [2])		Checklist	Yes	
7	Objective of the review	Objective	Describe the primary question and secondary questions (when applicable).	The primary question is the main question of the review. Secondary questions are usually linked to sources of heterogeneity (effect modifiers).	Checklist	Yes	
8		Definitions of the question components	Break down and summarise question key elements e.g. population, intervention(s)/exposure(s), comparator(s), and outcome(s).	For other question types see [3,4]	Meta-data	Population = All marine species;<br>Intervention = All GGI interventions applied to multifunctional marine structures. I.e. the addition, modification, or construction of ecological enhancements;<br>Comparator = One or more of the following options:<br>-Unmodified structure vs ecologically enhanced structure (i.e. structure with intervention applied)<br>-Natural environment vs ecologically enhanced structure<br>-Ecologically enhanced structure 1 (using Intervention A) vs ecologically enhanced structure 2 (using Intervention B)<br>-Structure Before vs After ecological enhancement;<br>Outcomes = Abundance, Biomass, Species diversity, Species composition, Functional group diversity	
	Methods						
9	Searches	Search strategy	Detail the planned search strategy to be used, including: database names accessed, institutional subscriptions (or date ranges subscribed for each database), search options (e.g. 'topic words' or 'full text' search facility), efforts to source grey literature, other sources of evidence (e.g. hand searching, calls for evidence/submission of evidence by stakeholders).	Details regarding search strategy testing should be provided.	Checklist	Yes	
10		Search string	Provide Boolean-style full search string and state the platform for which the string is formatted (e.g. Web of Science format)		Meta-data	(bioblock$ OR "bio* enhanc*" OR "bio* mimic*" OR biophilic OR complex* OR "eco-eng*" OR "ecoeng*" OR "eco* engineer*" OR "eco* enhance*" OR "eco-design*" OR "eco-innovation$" OR "eco-restor*" OR "eco-sensitive" OR "eco-tech*" OR "engineer* complex*" OR GGI OR GBGI OR "green eng*" OR greening NEAR/3 grey OR "habitat enhanc*" OR "habitat augment*" OR "habitat enhanc* panel$" OR "habitat modif*" OR "habitat panel$" OR "habitat tile$" OR heterogen* OR IGGI OR install* OR "intertidal mitigat*" OR "living shore*" OR "living seawall$" OR manipulat* OR microhabitat$ OR microtopograph* OR mimic* OR "nature-based solution$" OR "nature-inclu* design*" OR NbS OR rough* OR "structur* complex*" OR "substrat* enhanc*" OR textur* OR topograph* OR transplant*) AND (armor* OR armour* OR "anthropogenic structure$" OR "anthropogenic infrastructure" OR "artificial habitat$" OR "artificial infrastructur*" OR "artificial shore*" OR "artificial structur*" OR breakwater$ OR "built structure$" OR bulkhead$ OR caisson$ OR "coast* defen*" OR "coast* hard*" OR "coast* protect*" OR embankment$ OR "engineer* structur*" OR "coast* guard*" OR dike$ OR dock$ OR dolphin$ OR dyke$ OR "float* dock*" OR "float* structure*" OR "flood* defenc*" OR "grey infrastructure" OR "gray infrastructure" OR groin$ OR groyne$ OR humanmade OR "human-made" OR infrastructure OR jett* OR "manmade structure$" OR "man-made structure$" OR marina$ OR "marine construction$" OR "marine urban*" OR mooring$ OR "ocean sprawl" OR "offshore structur*" OR "rig$" OR pier$ OR piling$ OR pontoon$ OR port$ OR quay OR revetment$ OR riprap OR "sea defence$" OR seawall* OR "sea wall*" OR "shore* protect*" OR "storm surge barrier$" OR "tidal energy" OR tile$ OR "underwater structure$" OR "wave break*" OR wavescreen$ OR "wind turbine$" OR "oil and gas" OR "oil and gaz" OR "oil & gas" OR "oil & gaz" OR "petroleum installation*" OR "windfarm$" OR "wind farm$" OR MREI OR "marine renewable$" OR "wave farm*" OR "tidal energy" OR "tidal stream*") AND (coast* OR estuar* OR intertidal OR marine OR ocean* OR offshore OR subtidal OR shore* OR "water column" OR pelagic OR benthic) AND (assemblage$ OR diversit* OR abundance$ OR biodiversit* OR biomass* OR "community structure$" OR "community composition$" OR "functional composition$" OR "functional evenness" OR "functional group$" OR "functional richness" OR "functional trait composition" OR "species composition" OR "species evenness" OR "species presence" OR "species richness" OR "species similarit*" OR "taxonomic composition$" OR "taxonomic richness") NOT (oceanography OR "zostera marina" OR "bottlenose dolphin$")	Supplimentary File 3
11		Languages – bibliographic databases	List languages to be used in bibliographic database searches.		Meta-data	English	
12		Languages – grey literature	List languages to be used in organizational websites searches and web-based search engines.		Meta-data	English	
13		Bibliographic databases	Provide the number of bibliographic databases to be searched.		Meta-data	>3: Web of Science Core Collection, Scopus, databases within Proquest	
14		Web – based search engines	Provide the number of web – based search engines to be searched.		Meta-data	1: Google Scholar	
15		Organisational websites	Provide the number of organisational websites to be searched.		Meta-data	>20	
16		Estimating the comprehensiveness of the search	Describe the process by which the comprehensiveness of the search strategy was assessed (i.e. list of benchmark articles).		Checklist	Yes	
17		Search update	Describe any plans to update the searches during the conduct of the review.	Optional. A search update is good practice if original searches were performed more than two years prior to review completion.	Checklist	n/a	
18	Article screening and study inclusion criteria	Screening strategy	Describe the methodology for screening articles/studies for relevance/eligibility.		Checklist	Yes	
19		Consistency checking	Describe clearly the process for checking consistency of decisions including the levels at which consistency checking will be undertaken and estimated proportion of articles/studies that will be screened and checked for consistency by two or more reviewers (e.g. Titles (10%), abstracts (10%), full text (10%)).		Checklist	Yes	
20		Inclusion criteria	Describe the inclusion criteria used to assess relevance of identified articles/studies. These must be broken down into the question key elements (e.g. relevant subject(s), intervention(s)/exposure(s), comparator(s), outcomes, study design(s)) and any other restrictions (e.g. date ranges or languages).		Checklist	Yes	
21		Reasons for exclusion	State that you will provide a list of articles excluded at full text with reasons for exclusion.		Checklist	Yes	
22	Critical appraisal	Critical appraisal	Describe here the method you propose for critical appraisal of study validity (including assessment of individual studies and the evidence base as a whole).		Checklist	Yes	
23		Critical appraisal strategy	Describe how the information from critical appraisal will be used in synthesis.		Checklist	Yes	
24		Consistency checking	Describe how repeatability of critical appraisal of study validity will be tested.		Checklist	Yes	
25	Data extraction	Meta-data extraction and coding strategy	Describe the method for meta-data extraction and coding for studies (potentially providing forms/data sheets (ideally piloted), list if variables to be extracted as meta-data and those that will be coded).		Checklist	Yes	
26		Data extraction strategy	Describe the method for extraction of qualitative and/or quantitative study findings (potentially providing forms/data sheets (ideally piloted))		Checklist	Yes	
27		Approaches to missing data	Describe any processes for obtaining and confirming missing or unclear information or data from authors.		Checklist	Yes	
28		Consistency checking	Describe how repeatability of the meta-data/data extraction process will be tested.		Checklist	Yes	
29	Potential effect modifiers/reasons for heterogeneity	Potential effect modifiers/reasons for heterogeneity	Provide a list of and justification for the effect modifiers /reasons for heterogeneity that will be considered in the review. Also provide details of how the list was compiled (including consultation of external experts).	The list should not be exhaustive but a short list of those variables thought to be most important and amenable to analysis.	Checklist	Yes	
30	Data synthesis and presentation	Data synthesis and presentation	State the type of synthesis conducted as part of the systematic review (narrative only, narrative and quantitative, narrative and qualitative, narrative, qualitative and quantitative, narrative and mixed-methods)		Meta-data	Narrative synthesis and meta-analysis	
31		Narrative synthesis strategy	Describe methods to be used for narratively synthesising the evidence base in the form of descriptive statistics, tables (including any map databases) and figures.	Vote-counting (tallying of studies based on the direction or significance of their findings) must be avoided. Must include a summary of the outputs of critical appraisal of the evidence base as a whole.	Checklist	Yes	
32		Quantitative synthesis strategy	If data are appropriate for quantitative synthesis, describe planned methods for calculating effect sizes, methods for handling complex data, statistical methods for combining data from individual studies, and any planned exploration of heterogeneity (e.g. sensitivity analysis, subgroup analysis and meta-regression). If all studies may not be selected for synthesis explain criteria for selection (e.g. incomplete or missing information).	Compulsory if appropriate for data	Checklist	Yes	
33		Qualitative synthesis strategy	Describe methods to be used for synthesising qualitative data and justify your methodological choice. Describe if and how you plan to analyse subgroups/subsets of data. If all studies may not be selected for synthesis explain criteria for selection (e.g. incomplete or missing information).	Compulsory if appropriate for data	Checklist	n/a	
34		Other synthesis strategies	Describe any other approaches to be used for synthesising data or combining qualitative and quantitative synthesis (e.g. mixed-methods) and justify your methodological choice.	Compulsory if appropriate for data	Checklist	n/a	
35		Assessment of risk of publication bias	Describe planned methods for examining the possible influence of publication bias on the synthesis.	For quantitative syntheses this may be done using diagnostic plots or statistical tests	Checklist	Yes	
36		Knowledge gap identification strategy	Describe the methods to be used to identify and/or prioritise key knowledge gaps (unrepresented or underrepresented subtopics that warrant further primary research).	Optional	Checklist	n/a	
37		Demonstrating procedural independence	Describe the role of systematic reviewers (who have also authored articles to be considered within the review) in decisions regarding inclusion or critical appraisal of their own work.	Reviewers who have authored articles to be considered within the review should be prevented from unduly influencing inclusion decisions, for example by delegating tasks appropriately.	Checklist	Yes	
38	Declarations	Competing interests	Describe of any financial or non-financial competing interests that the review authors may have.		Checklist	Yes	

References

[1] Bayliss, H.R., Haddaway, N.R., Eales, J., Frampton, G.K. and James, K.L., 2016. Updating and amending systematic reviews and systematic maps in environmental management. Environmental Evidence, 5(1), p.20.

[2] Haddaway, N.R., Kohl, C., da Silva, N.R., Schiemann, J., Spök, A., Stewart, R., Sweet, J.B. and Wilhelm, R., 2017. A framework for stakeholder engagement during systematic reviews and maps in environmental management. *Environmental Evidence* , *6* (1), p.11.

[3] Collaboration for Environmental Evidence. 2018. Guidelines and Standards for Evidence synthesis in Environmental Management. Version 5.0. www.environmentalevidence.org/information-for-authors.

[4] Leeds Institute of Health Sciences. https://medhealth.leeds.ac.uk/info/639/information_specialists/1500/search_concept_tools. Accessed 12/11/2017.
